# Improving speech depression detection using transfer learning with wav2vec 2.0 in low-resource environments

**DOI:** 10.1038/s41598-024-60278-1

**Published:** 2024-04-25

**Authors:** Xu Zhang, Xiangcheng Zhang, Weisi Chen, Chenlong Li, Chengyuan Yu

**Affiliations:** 1 School of Software Engineering, Xiamen University of Technology, Xiamen, 361024 China; 2 School of Computer and Information Engineering, Xiamen University of Technology, Xiamen, 361024 China; 3 School of Computer and Information Engineering, Jiangxi Agricultural University, Nanchang, 330045 China

**Keywords:** Computational science, Diagnosis

## Abstract

Depression, a pervasive global mental disorder, profoundly impacts daily lives. Despite numerous deep learning studies focused on depression detection through speech analysis, the shortage of annotated bulk samples hampers the development of effective models. In response to this challenge, our research introduces a transfer learning approach for detecting depression in speech, aiming to overcome constraints imposed by limited resources. In the context of feature representation, we obtain depression-related features by fine-tuning wav2vec 2.0. By integrating 1D-CNN and attention pooling structures, we generate advanced features at the segment level, thereby enhancing the model's capability to capture temporal relationships within audio frames. In the realm of prediction results, we integrate LSTM and self-attention mechanisms. This incorporation assigns greater weights to segments associated with depression, thereby augmenting the model's discernment of depression-related information. The experimental results indicate that our model has achieved impressive F1 scores, reaching 79% on the DAIC-WOZ dataset and 90.53% on the CMDC dataset. It outperforms recent baseline models in the field of speech-based depression detection. This provides a promising solution for effective depression detection in low-resource environments.

## Introduction

Depression, a widespread mental disorder, significantly jeopardizes individual well-being^1^. In the aftermath of the COVID-19 pandemic, the global impact of mental disorders has become more evident. According to estimates from the World Health Organization (WHO)^1^, this pandemic has led to a 25%-27% increase in the global prevalence of depression and anxiety. While efficient treatments for mental illnesses exist, a substantial majority of patients in lower and middle-income nations lack proper access to healthcare^3^. Moreover, a primary approach to screening for depression involves the use of diagnostic scales and psychiatric interviews. However, societal stigma and unequal distribution of medical resources contribute to a generally high recurrence rate of depression. Research findings suggest that timely identification and support for individuals at risk of depression can effectively reduce the likelihood of developing depression^4^. Therefore, exploring an automated and cost-effective method for detecting depression with universal applicability is of utmost significance.

In recent years, researchers have collected biometric information related to depression, encompassing speech^5^, facial behavior^6^, and text^7^, utilizing convenient and accessible sensing devices. They advocate the utilization of machine learning approaches for depression detection, emphasizing the non-invasiveness and objectivity inherent in these methods. Notably, speech features have demonstrated a close correlation with the severity of depression^8^. In practical applications, speech signals offer greater accessibility and privacy compared to other behavioral signals, making them widely applied in emotion recognition^9,10^. Consequently, an increasing number of researchers are exploring Speech Depression Detection (SDD) via the utilization of advanced computational techniques such as machine learning and deep learning. Nevertheless, persistent technical challenges persist in the development of SDD models.

Firstly, deep learning training typically relies on substantial annotated data to achieve satisfactory classification performance. However, acquiring annotated data in the field of depression poses challenges due to concerns about patient privacy^11^. Moreover, due to the specialized nature of the medical field, non-experts find it challenging to make accurate judgments about speech. This often necessitates substantial time investments from medical professionals in data processing. The progress of SDD is notably impeded by the scarcity of resources, significantly impacting both the model's performance and its ability to generalize^12^. To address the issue of limited resources, contemporary approaches incorporate techniques such as data augmentation^13^, meta-learning^14^, and transfer learning^15,16^. For instance, pre-trained models like BERT have demonstrated remarkable performance in text-based depression detection through transfer learning. Similarly, wav2vec, a pre-trained model in the audio domain, exhibits excellence and holds the potential for transfer learning to diverse domains. However, its application in SDD remains relatively limited at present.

Furthermore, in the diagnosis of depression, a single diagnostic result is derived from multiple rounds of dialogue without providing detailed labels for each specific time point^17^. This challenge necessitates maintaining integrity when handling temporal information. It involves focusing on relevant information from lengthy conversations and avoiding interference from redundant information. To address the constraint of model input size, prior research^18–20^ segmented audio, modeling information within each audio segment, yet overlooking the temporal dynamics of the entire audio sequence. Therefore, Du et al.^21^ partitioned each speech segment into 7-second intervals, extracting Mel-frequency cepstral coefficients (MFCC) and Linear Predictive Coding (LPC) features for each segment. They subsequently employed a combination of a one-dimensional convolutional neural network (1D-CNN) and Long Short-Term Memory (LSTM) to classify depression-related features both within and between speech segments. Although this method restricts the model's input size and retains input information from the entire audio segment, the performance of LSTM models may be suboptimal in handling long-time sequences.

Finally, a notable concern arises regarding the potential loss of temporal information between audio frames. Regardless of the segmentation strategy employed, audio segments must be subdivided into frames, necessitating the conversion of frame-level features to paragraph-level features. In previous studies^22^, prevalent strategies involved maximum pooling or average pooling. However, these methods exhibit a limitation in failing to preserve the temporal information inherent between frames.

To address the aforementioned challenges, we introduce a novel model designed specifically for depression classification in speech. The proposed model comprises four key steps. Initially, raw audio undergoes preprocessing through segmentation, eliminating irrelevant segments. Subsequently, advanced features are extracted through the fine-tuning of the wav2vec 2.0 model. Thirdly, a 1D-CNN+attention pooling structure is employed to encode frame-level features of the speech, yielding sentence-level feature representations. Finally, depression classification is executed using a combination of Long Short-Term Memory (LSTM) and self-attention mechanisms. We applied the proposed method to a genuine diagnostic dataset and compare the classification results with those of existing methods. On the same small sample dataset, our method outperforms existing approaches without the need for additional data augmentation strategies.

The contributions of this paper are threefold:

- To address the challenge of low-resource data, we advocate for transfer learning on the wav2vec 2.0 model, employing it as audio feature input for downstream models. In comparison to existing methods, we utilize only a single-class feature and achieve superior performance. To the best of our knowledge, this marks the inaugural proposal of fine-tuning the wav2vec 2.0 model to specifically tackle the low-resource challenge in SDD.
- We introduce a strategy based on 1D-CNN + attention pooling to enhance the feature representation capability within speech segments. Based on the results of downstream tasks, the proposed structure in this paper more effectively captures the temporal relationships between frames compared to statistical functions (e.g., maximum pooling, average pooling). As a result, it produces a more expressive segment-level vector representation for depression assessment tasks.
- Through the incorporation of a self-attention mechanism into the downstream output of LSTM, we successfully mitigate interference from irrelevant speech segments, leading to a notable enhancement in the overall recognition capability.

## Related work

In this section, we will discuss relevant works on speech depression detection and transfer learning.

### Depression detection based on audio

Numerous methods suitable for SDD have been proposed, primarily consisting of two components: speech feature extraction and model construction. In the early studies on audio-based depression detection, the focus was on manual speech feature extraction. After feature extraction, machine learning classification algorithms were applied to explore the relationship between features and the severity of depression. For instance, Naulegari Janardhan et al.^23^ introduced a feature selection algorithm based on Fisher scores. This algorithm dynamically integrates the selection of acoustic features, thereby enhancing the accuracy of depression prediction. Kaur B et al.^24^ presented a feature selection method based on the Quantum Whale Optimization Algorithm to choose minimally correlated and non-redundant speech features. Their approach, utilizing a fusion of temporal, spectral, and spectro-temporal features, demonstrated optimal performance in an LDA classifier. While manual feature extraction has shown some effectiveness in depression detection, it often requires considerable expertise to select appropriate tools for extracting feature sets, and there may be issues of feature redundancy in the feature set.

With the revolutionary progress of deep learning technology in automatic feature extraction and classification, it excels in extracting high-level semantic features, demonstrating strong adaptability and transferability compared to machine learning methods. Furthermore, it is demonstrated to be more dependable and efficient in extracting depression-related features when compared to traditional manual feature extraction techniques^25^. Lu et al.^19^ introduced a model that combines a Transformer Encoder and CNN utilizing the former to capture temporal information and the latter to extract high-level speech features, ultimately facilitating the prediction of depression severity. To increase the sample size and avoid excessively long input sequences, they divided each response into multiple segments, each lasting 3 s with a 50% overlap. Miao et al.^20^ divided speech into 4-s segments, using a combination of high-order spectral analysis and the fusion of traditional speech features. They employed classification models such as CNN. Zhou et al.^26^ proposed a depression detection model centered on the segmentation of question–answer-level speech data segmentation and hierarchical multi-feature fusion. The primary objective was to diminish the size and complexity of the model, and they achieved good performance. However, it is worth noting that despite the good results achieved in speech segments or their combinations in the aforementioned studies, no testing was conducted on the entire speech. Although Du et al.^21^ employed an LSTM model to extract temporal-related features between speech segments, LSTM may still face challenges in capturing long-term dependencies in audio signals. Zhang et al.^17^ introduced a self-supervised audio feature extraction method called DEPA, which learns high-level representations of audio by reconstructing the central part of the spectrogram.

Generally, the studies mentioned above are hindered by the challenge of limited resources. In other words, despite ensuring a sufficient quantity of data, they are unable to fully exploit the experimental potential of the entire audio segment information.

### Transfer learning

To tackle the previously mentioned issue of low resources, in addition to data augmentation, transfer learning^27^ proves to be an effective method. This involves training a model in the source domain and transferring the acquired knowledge to the target domain, thereby enhancing the performance of the target task and addressing the issue of data scarcity in the target domain. Huang et al.^28^ utilized two depression speech datasets collected in different environments and proposed a depression detection framework based on a convolutional neural network and channel coordination information. They utilized three different transfer learning strategies, including layer-wise adaptation and cumulative adaptation (from front to back or from back to front), to enhance the generalization ability across different corpora. Rejaibi et al.^29^ proposed a deep neural network model utilizing MFCC features and LSTM. Through pretraining and fine-tuning on a related task of emotion recognition, they effectively enhanced the recognition and assessment capabilities of depression, particularly in identifying depression in females. Besides pretraining on datasets in similar domains, leveraging large models trained with self-supervised learning is also a viable choice.

In practical applications, choosing large models trained with self-supervised learning can offer rich speech representations for depression detection tasks. This approach involves pretraining on unlabeled speech data by automatically generating labels, thus learning more generalized feature representations. Following pretraining, fine-tuning the model for a specific depression detection task helps enhance its performance in the target domain. Pepino et al.^30^ introduced a method that leverages features from various layers of the pretrained wav2vec 2.0 model and a trainable weighted average layer for speech emotion recognition tasks, achieving significant performance improvement. The study also found that fusing features of the wav2vec 2.0 model with a set of prosodic features can result in additional performance improvement. Through the utilization of CNN and pretrained models such as Wav2Vec 2.0 and BERT to model both speech and language, a study^31^ observed that speech demonstrates a greater capacity to differentiate Parkinson's disease patients compared to language. Chen et al.^32^, with the limited DiCOVA dataset, achieved good results in COVID-19 diagnosis tasks by combining supervised and unsupervised pretraining methods, using the wav2vec 2.0 model to extract high-level features. Nowakowski et al.^33^ emphasized that in situations where labeled data for the target language is exceedingly scarce, fine-tuning a pretrained speech representation model (such as wav2vec 2.0) trained on multiple languages can significantly enhance its performance in speech transcription tasks.

The above-mentioned studies have conclusively demonstrated the superiority of transfer learning, providing more robust feature representations for tasks with limited samples. Therefore, we are investigating ways to optimize the performance of the wav2vec 2.0 model in SDD to tackle the low-resource challenge.

## Materials and methods

### Problem definition

We denote the raw speech data of the $i$-$th$ participant in the dataset as the variable ${x}_{i}$. Each variable $x$ corresponds to a real state label ${y}_{i}$, where ${y}_{i}$ belongs to the set $\{\mathrm{0,1}\}$, with ${y}_{i}$=0 indicating normal and ${y}_{i}$=1 indicating depression. Our goal is to utilize deep learning techniques to extract features related to depression from ${x}_{i}$ and predict the depression status ${y}_{i}$ for each participant.

**Figure 1:**
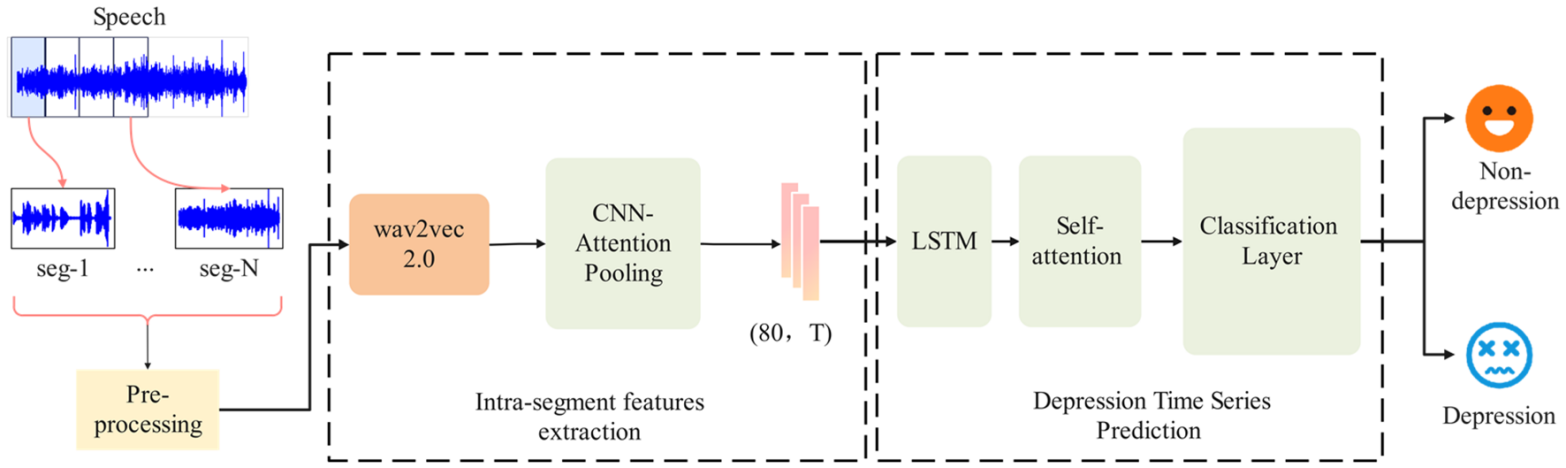
The proposed model's framework consists of three main components: (1) preprocessing, segmenting the audio signal into fixed time intervals; (2) Intra-segment feature extraction, extracting frame-level features from wav2vec in each segment, which undergo one-dimensional convolution and attention pooling for enhanced representations; (3) individual-level depression prediction for each segment using LSTM and self-attention mechanisms based on learned features.

The proposed method framework, illustrated in Fig. 1, comprises four key steps: audio preprocessing, frame-level feature extraction, segment-level feature extraction, and depression classification. The proposed model's framework consists of three main components:(1) preprocessing, segmenting the audio signal into fixed time intervals; (2) Intra-segment feature extraction, extracting frame-level features from wav2vec in each segment, which undergo one-dimensional convolution and attention pooling for enhanced representations; (3) individual-level depression prediction for each segment using LSTM and self-attention mechanisms based on learned features. Initially, we segment the preprocessed audio signal ${x}_{i}$ into fixed time lengths. Assuming '$M$' sections in each audio segment, the segmentation is denoted as ${x}_{i}=\{{s}_{i,1},...,{s}_{i,j},...,{s}_{i,M}\}(j\in [1,M]$), where ${s}_{i,j}$ represents the jth speech segment after preprocessing for the ith subject. Subsequently, we extract frame-level features from each segment ${s}_{i,j}$. Assuming '$N$' frames in each speech segment, it can be expressed as ${s}_{i,j}=\{{h}_{1},\cdots ,{h}_{n},\cdots ,{h}_{N}\}$($n\in [1,N]$), where ${{\text{h}}}_{{\text{n}}}$ signifies the feature vector of the nth frame in the segment, with each frame possessing 'd' dimensions. Thus, ${s}_{i,j}$ is a two-dimensional matrix of $n\times d$. Following this, a convolutional neural network is employed to derive paragraph-level advanced features ${c}_{i,j}$, which are then compressed into a one-dimensional feature vector ${v}_{i,j}$ through a pooling layer. At this stage, each subject is represented as ${x}_{i}=\{{v}_{i,1},{v}_{i,2},...,{v}_{i,j}\}$, where ${v}_{i,j}$ denotes the final advanced feature representation of each segment. Finally, we construct a temporal prediction model utilizing LSTM and a self-attention mechanism. Leveraging the features from all segments for each subject, we predict the mental state ${y}_{i}$. The aforementioned definitions will maintain consistency throughout this method, and needless repetition shall be avoided.

### Speech preprocessing

In this study, our focus is exclusively on analyzing the speech of the subjects, with the exclusion of all non-subject segments, including the interviewer's voice, silent intervals, and background noise. The entire speech corpus is then partitioned into fixed-duration, non-overlapping segments while preserving the original temporal sequence. Based on prior research outcomes^21,34^, we determine the optimal segment length to be 7 s through experimental enumeration, a conclusion that aligns with our experimental findings. When handling speech sequence data, a common preprocessing step involves standardizing the segment length. This not only establishes a consistent input size for the model, thereby enhancing computational efficiency, but also augments the number of training samples. Moreover, in comparison to methods reliant on semantic content for segmentation in text, this approach is more succinct, necessitating no additional trimming or superfluous operations. As a result, it maximizes the inclusion of all speech segments from the subjects.

### Segment-level feature extraction

#### Based on wav2vec 2.0 frame-level feature extraction

**Figure 2:**
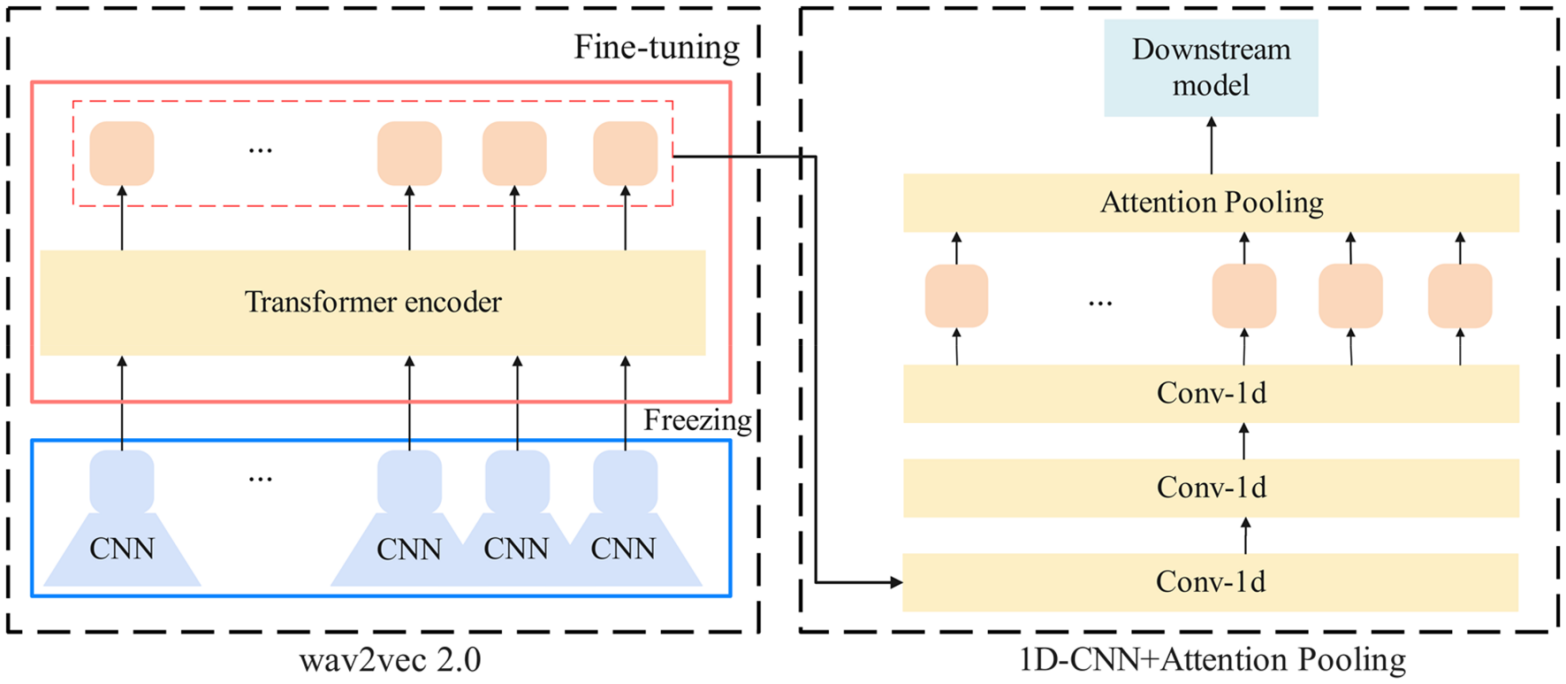
The framework for intra-segment feature extraction is structured as follows: on the left side, the pre-trained model of wav2vec2.0 is depicted, with frozen encoding layers and fine-tuning employed for the decoding layers. On the right side, a combination of 1D-CNN and attention pooling layer is utilized to discern the varying significance of depression-related information present in different frames of speech.

Wav2vec 2.0^35^ stands as a self-supervised learning framework specifically crafted for extracting robust representations from raw speech signals. The fundamental concept underlying Wav2vec 2.0 involves formulating self-supervised training objectives through vector quantization, extensive input masking, and the utilization of a contrastive learning loss function during training. The architectural representation of the model is illustrated in Fig. 2. The model takes segmented speech sequence fragments ${\{s}_{i,1},...,{s}_{i,j},...,{s}_{i,M}\}$ from the original audio and feeds them into a multi-layer convolutional feature encoder. This encoder transforms the input fragments into latent speech representations with a frame length of 25 ms and a frame shift of 20 ms, resulting in ${\{Z}_{1},{Z}_{2},\cdots ,{Z}_{T}\}$. Consequently, all audio data in this study is upsampled to 16 kHz to adhere to the input requirements of wav2vec 2.0.Subsequently, the latent representations are input into the context encoder, which captures sequential information and outputs the final speech representations $\{{h}_{1},\cdots ,{h}_{n},\cdots ,{h}_{N}\}$. The context encoder comprises multiple layers of Transformer encoders, categorized into the base model (12 layers) and the large model (24 layers) based on the number of layers employed. The model achieves commendable performance through pretraining on a substantial volume of unlabeled speech data, followed by fine-tuning on annotated speech data tailored to specific tasks.

In this investigation, we perform a comparative analysis between the original Wav2vec 2.0 base model and the Wav2vec 2.0 large model. Our approach involves inputting preprocessed speech data into both models and separately fine-tuning the last layer and all layers to derive the final speech representations. Fine-tuning of the Transformer layers within the Wav2vec 2.0 network is carried out while maintaining the integrity of the lower convolutional layers. Subsequently, the output of all Transformer layers for each audio segment is aggregated by summation, yielding the Wav2vec 2.0 feature sequence for the audio. This process ensures that the contributions from each Transformer layer are integrated to produce a comprehensive representation of the speech content in the audio segment.

Moreover, a significant association exists between depression and emotions. Wu et al.^36^ investigated depression detection by employing pretraining features derived from an emotion recognition model. Their proposed approach, termed emotion transfer, notably enhanced the model's performance in detecting depression. Motivated by this study, we conducted comparative experiments by utilizing a model fine-tuned on the IEMOCAP emotional dialogue dataset.

#### 1D-CNN and attention pooling layer

To generate sophisticated feature representations of speech signals, we employed an acoustic feature extraction method that integrates both Convolutional Neural Network and self-attention pooling. This approach is designed to yield high-level representations of speech signals. The detailed network structure is depicted in the right portion of Fig. 2.

Initially, we established a sequential model aimed at extracting advanced features from each frame of the audio. This model consists of three convolutional blocks, each composed of a 1-D convolutional layer, a ReLU activation function, and a dropout layer. The number of filters for these convolutional layers is individually set to $C=[\mathrm{80,80,80}]$. To introduce non-linearity and address overfitting, each convolutional layer is succeeded by ReLU and a random dropout layer. The input dimension of the convolutional layers corresponds to the audio feature dimension, while the output dimension is pre-defined as the hidden layer dimension. We incorporated an average pooling layer to extract higher-level features by reducing the time dimension. The convolutional layers execute convolution operations by sliding a fixed-size window over the input data, extracting features within the window, and mapping these features to the subsequent layer. For each speech sequence segment ${s}_{i,j}=\{{h}_{1},\cdots ,{h}_{n},\cdots ,{h}_{N}\}$, we applied one-dimensional convolutional operations, yielding the advanced feature sequence ${C}_{i,j}=\{{h}_{1}{\prime},\cdots ,{h}_{n}{\prime},\cdots ,{h}_{N}{\prime}\}$, as depicted in Eq. (1).

1 $${C}_{i,j}=conv1D({s}_{i,j},K)\in {R}^{T\times d}$$

where conv1d represents the one-dimensional convolution function, $K$ represents the size of the convolutional kernel, resulting in the output tensor $C$, $d$ signifies the compressed feature dimension, and $T$ denotes the duration of the speech.

Subsequently, acknowledging that each frame of every audio segment encapsulates distinct information, pooling operations become instrumental in extracting comprehensive insights from frame-level features. Consequently, we introduced pooling layers to derive global features across speech segments. At this juncture, we evaluated three distinct pooling methods: max pooling, average pooling, and attention pooling. These methods contribute to capturing essential information in the audio sequence, thereby augmenting the expressiveness of the features.

Average Pooling: For each audio segment ${C}_{i,j}$, the feature values of all frames within this audio are summed and then divided by the number of frames to yield the average value, serving as the feature representation for each audio segment, as illustrated in Eq. (2).

2 $${AveragePooling}_{{C}_{i,j}}=\frac{1}{N}{\sum }_{n=1}^{N}{h}_{n}{\prime}$$

Max pooling: For each audio segment ${C}_{i,j}$, select the maximum value of the feature values of all frames on this audio as the feature of each audio segment, as indicated in Eq. (3).

3 $${MaxPooling}_{{C}_{i,j}}={max}_{n=1}^{N}{h}_{n}{\prime}$$

Attention Pooling: The incorporation of an additive attention mechanism enhances the network's focus on significant frames within the audio, thereby boosting feature expressiveness in the pooling process. For each audio segment ${C}_{i,j}$, a weighted sum of frame-level features is conducted to generate the ultimate feature representation for that audio segment. This process dynamically adjusts weights in the time series to accentuate essential contextual information. Given an encoded sequence ${C}_{i,j}=\{{h}_{1}{\prime},\cdots ,{h}_{n}{\prime},\cdots ,{h}_{N}{\prime}\}$, the speech-level feature representation ${V}_{i,j}$ is computed using the following formula:

4 $${V}_{i,j}=Softmax({w}_{c}{{C}_{i,j}}^{T}){C}_{i,j}$$

where the matrix ${w}_{c}$ represents a learnable weight matrix, dynamically capturing the significance of each frame feature through weighted averaging to derive the final speech-level feature. This adaptive mechanism enables the network to focus more effectively on segments of the speech deemed critical for the task, resulting in feature vectors with heightened semantic expressiveness. Ultimately, the final feature representation is fed into the downstream network to assimilate global temporal information.

### Depression prediction model incorporating temporal information

The LSTM model we have established adeptly captures both short-term and long-term temporal correlations between segments across the entire dialogue. This is achieved through the orchestrated interplay of the forget gate, input gate, and output gate within LSTM. These components effectively manage the neuron state, ensuring the orderly transmission of relevant sequence information. Moreover, LSTM addresses challenges such as gradient explosion and gradient disappearance, which can arise when dealing with lengthy time series^37^. While LSTM demonstrates commendable predictive capabilities for the temporal dynamics of time series data in SDD, it encounters the potential challenge of forgetting early learning content in the context of long sequence samples. This could lead to the loss of crucial information, ultimately impacting predictive accuracy. The input for each participant ${x}_{i}$ based on the LSTM-based time series extraction network is outlined as follows:

5 $${x}_{i}{\prime}=lstm({[v}_{i,1},{v}_{i,2},...,{v}_{i,j}])$$

**Figure 3:**
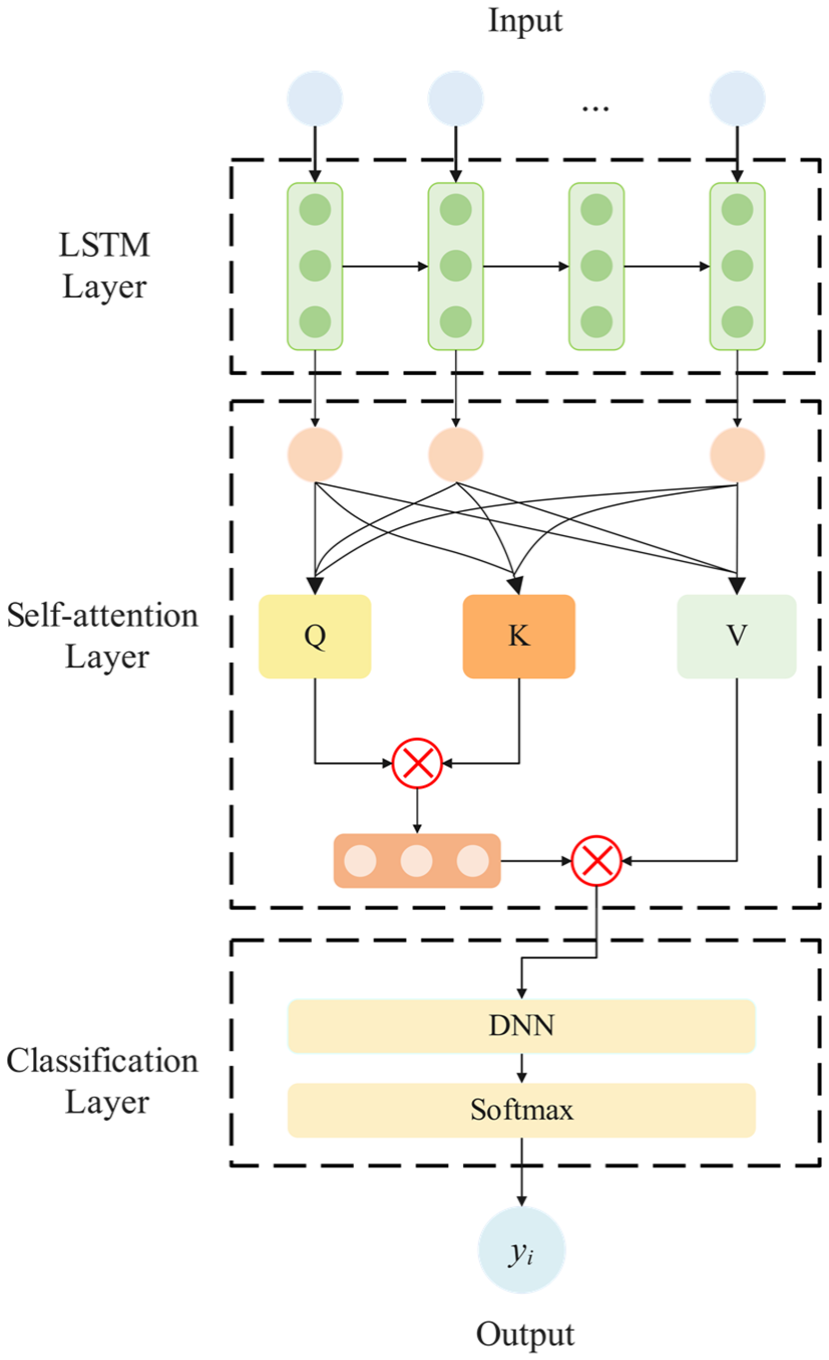
Temporal depression prediction model between inter-segment based on LSTM and self-attention mechanism.

The Self-Attention Mechanism^38^ proves to be a potent tool for capturing dependencies among different segments within extended dialogue sequences. It excels at assigning distinct attention weights to individual speech features, thereby enhancing the model's comprehension of the depressive tendencies embedded in the entire conversation. Introducing the Self-Attention Mechanism effectively underscores the pivotal features influencing the prediction outcomes of depressive emotions. For the output sequence ${x}_{i}{\prime}=\{{v}_{i,1}{\prime},{v}_{i,2}{\prime},...,{v}_{i,j}{\prime}\}$ from the LSTM model, three matrices are derived through linear transformations, specifically the Query vector '$Q$', Key vector '$K$' and Value vector '$V$'. The interplay among these vectors is calculated to yield the weight output, and the calculation process is expressed as follows:

6 $$SelfAttention(x_{i}^{\prime } ) = Softmax\left( {\frac{{QK^{T} }}{{\sqrt {d_{K} } }}} \right)V$$

where, ${d}_{K}$ represents the dimension of '*K*' and the Softmax function is applied to normalize the weights within the range [0,1]. The resulting context vector ${x}_{i}{\prime}$ is obtained through the given equation and shares the same size as the input ${x}_{i}$. Subsequently, the sum of the values of ${x}_{i}{\prime}$ is fed into the classification layer, undergoes a linear transformation, and produces the binary classification result ${y}_{i}{\prime}$. The model architecture of this segment is illustrated in Fig. 3, encompassing input segment-level features, an LSTM layer, a self-attention mechanism layer, and a classification layer.

## Result

### Datasets description

The dataset employed in this study is the widely used Distress Analysis Interview Corpus with Wizard-of-Oz (DAIC-WOZ)^39^ and CMDC^40^.

DAIC-WOZ dataset comprises 189 clinical interviews meticulously crafted to facilitate the diagnosis of psychological distress conditions, including anxiety, depression, and post-traumatic stress disorder. The dataset is divided into a training subset (107 interviews), a development subset(35 interviews), and a test subset(47 interviews), amounting to a total of 50 h of data. The majority of prior studies conduct validation on the development set. For the sake of result comparison, our experiments are conducted on both the training subset and the validation subset. The collected data is multimodal, encompassing text, images, and speech information, with a focus on utilizing speech information as the experimental data. Each speech segment has an average length of 15 min, and a consistent sampling rate of 16 kHz is maintained throughout the dataset.

The CMDC dataset is a clinical depression dataset based on confirmed cases in Chinese language corpus, aiming to support screening and assessment of severe depression in China. This dataset also includes semi-structured interviews covering visual, auditory, and textual features. Unlike the DAIC-WOZ dataset, the CMDC dataset has predetermined twelve fixed questions during the interview. The CMDC dataset consists of 78 samples, including 26 cases of severe depression patients and 52 healthy individuals. Compared to DAIC-WOZ, the CMDC dataset is smaller in scale, further highlighting the scarcity of depression data.

### Evaluation metrics

Each participant contributes PHQ-8 scores, along with dichotomous labels. The PHQ-8 score indicates the degree of depression for each subject, while dichotomous labels signifies whether the subject is classified as depressed. The central aim of this paper is to predict whether the subject is a depression patient. Consequently, the evaluation metrics utilized in this study include accuracy (P), recall (R), and F1 score, area under the curve (AUC). The higher the value, the better the performance.

### Experimental settings

All experiments were executed on the Linux operating system, utilizing an NVIDIA V100 GPU, and implemented using the PyTorch framework. To optimize the fine-tuning of the audio pre-training model, we employed a small learning rate of 1e-5, while a learning rate of 0.006 was utilized for downstream tasks. The optimization process involved using an Adam optimizer with a weight decay of 0.001. The batch size of the training subset was set to 32, and the number of training epochs was defined as 200. The training process featured an automatic termination mechanism, activated when the model's performance on the validation set showed no significant improvement over 10 consecutive training epochs. Additionally, we employed the OpenSMILE tool to extract the IS09 emotion acoustic feature set. This set comprises 16 low-level descriptors (LLDs), such as Mel-frequency cepstral coefficients and zero-crossing rate, resulting in 32 LLDs computed by first-order differences. Subsequently, 12 statistical functions were applied to these descriptors to derive a 384-dimensional sentence-level feature representation. We utilized this representation for comparison with our fine-tuned features.

### Comparison with other methods

In this section, we conducted experiments comparing the DAIC and CMDC datasets in two different languages, as well as comparing the effects of different input features under the same model, to verify the robustness and effectiveness of our approach.

#### Performance evaluation on the DAIC-WOZ dataset

Table 1 presents a comprehensive comparison of our proposed method with recent approaches for depression detection based on speech, particularly on the DAIC-WOZ dataset. Our method achieves superior performance in terms of precision and F1 score, attaining values of 84.49% and 79.00%, respectively. In contrast to methods such as Chlasta et al.^41^, who generates additional training samples by cutting and randomly sampling audio files, and Rejaibi et al.^29^, who adopts a transfer learning strategy by pretraining on the RAVDESS database, our approach surpasses them, showcasing enhanced performance. Moreover, Othmani et al.^42^ address sparse data issues through audio augmentation techniques, yet our model outperforms them significantly, exhibiting an average 16.62% higher F1 score. This superiority is attributed to our use of the more generalizable pretraining model, wav2vec2.0, extensively trained on large-scale datasets, enabling more accurate capture of key features in speech data. Comparisons with Ravi et al.^43^, who use the Wav2vec2.0 model as a feature extractor and employ adversarial learning, demonstrate our model's outperformance in F1 score by 9.8%. This underscores the effectiveness of fine-tuned features in enhancing performance.

**Table 1:** A comparison of the proposed method with other methods for SDD on DAIC-WOZ dataset. Boldface highlights the highest score.

Method	Feature	Precision	Recall	F1-score
ResNet^41^ (2019)	spectrogram	57.14%	57.14%	57.14%
LSTM^29^ (2022)	MFCC	73.50%	64.50%	64.00%
EmoAudioNet^42^ (2021)	MFCC + Spectrogram	-	-	66.00%
DepAudioNet^43^ (2022)	wav2vec 2.0	-	-	69.20%
MSCDR^21^ (2023)	LPC + MFCC	66.70%	66.70%	74.60%
CNN + Channel-wise Attention^26^ (2022)	MFCC + Spectrogram + eGeMAPs	71.00%	**83.00%**	77.00%
Ours	is09_emotion	79.60%	68.66%	70.09%
	wav2vec 2.0	**84.49%**	76.99%	**79.00%**

**Figure 4:**
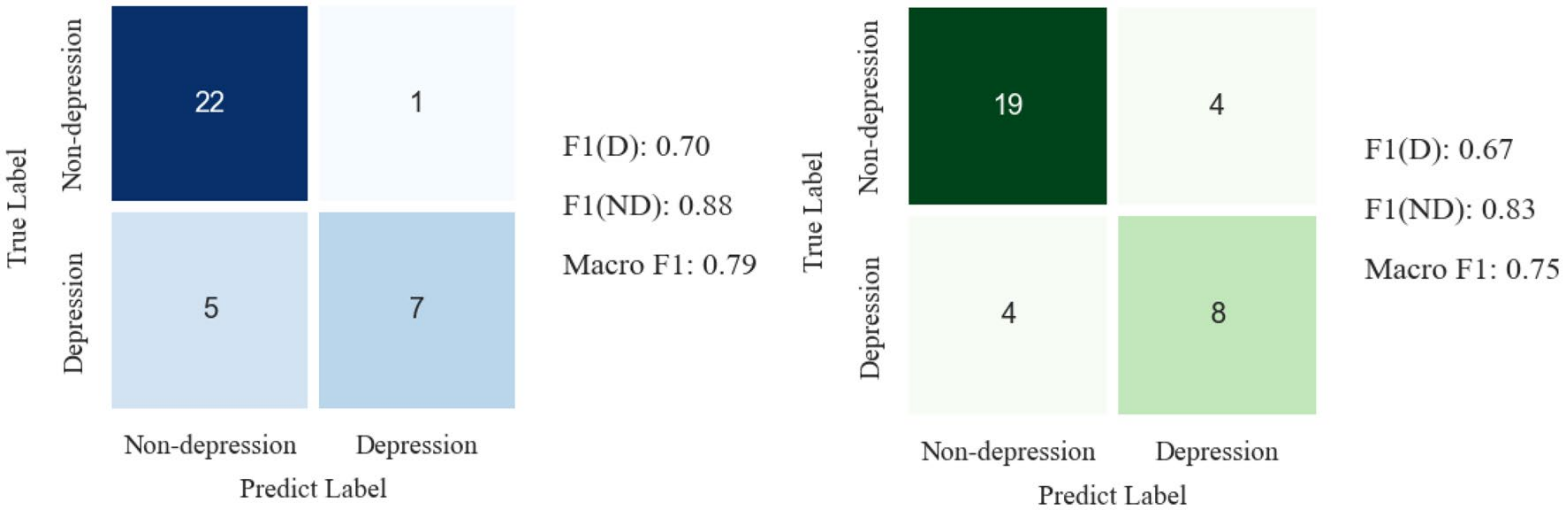
Comparative analysis of confusion matrices in depression detection: a comprehensive evaluation between the present study (left side) and DU et al. (right side). ND represents non-depression and D represents depression.

In contrast to Du et al.^21^, who extract MFCC and LPC features and use 1D-CNN and LSTM, our similar structure achieves significant improvements in precision, recall, and F1 score, outperforming them by 17.79%, 10.29%, and 4.4%, respectively. Examining the confusion matrix in Fig. 4 reveals a notable pattern: our model exhibits a higher count of true positives, contrasting with the comparator model that demonstrates a higher occurrence of false positives. This distinction suggests that our model is more discerning, effectively distinguishing non-depressive states. This increased discriminative ability enhances the model's reliability for practical applications, contributing to a heightened early detection rate for patients. This highlights the effectiveness of introducing wav2vec2.0 features, addressing the low-resource challenge, and incorporating a self-attention mechanism into the LSTM model to enable the model to ignore redundant information. Finally, despite Zhou et al.^26^ achieving the highest recall of 83% through the fusion of various descriptors, BoAW, functional features, and spectrograms, their precision and F1 score fall below our model's performance. Their segmentation approach sacrifices temporal information of the dialogue, while our model successfully retains richer long-term information, resulting in superior precision and F1 score.

#### Performance evaluation on the CMDC dataset

Table 2 presents the comparison results of our proposed method with recent speech-based depression detection methods on the CMDC dataset. Our method achieved the best performance in terms of precision and F1 score, reaching 94.83% and 90.53%, respectively. Compared to methods using acoustic prosodic features extracted from IS09, our precision increased by 12.51%, recall increased by 10.16%, and F1 score increased by 10.17%.

**Table 2:** A comparison of the proposed method with other methods for SDD on CMDC dataset.

Method	Feature	Precision (%)	Recall (%)	F1 (%)
Unsupervised encoder + Transformer^44^ (2022)	MFCC	92.00	83.00	87.00
OURS	is09_emotion	82.31	79.17	80.36
	Wav2vec 2.0	**94.83**	**88.33**	**90.53**

Boldface highlights the highest score.

### Comparing the binary classification performance of different acoustic features

**Figure 5:**
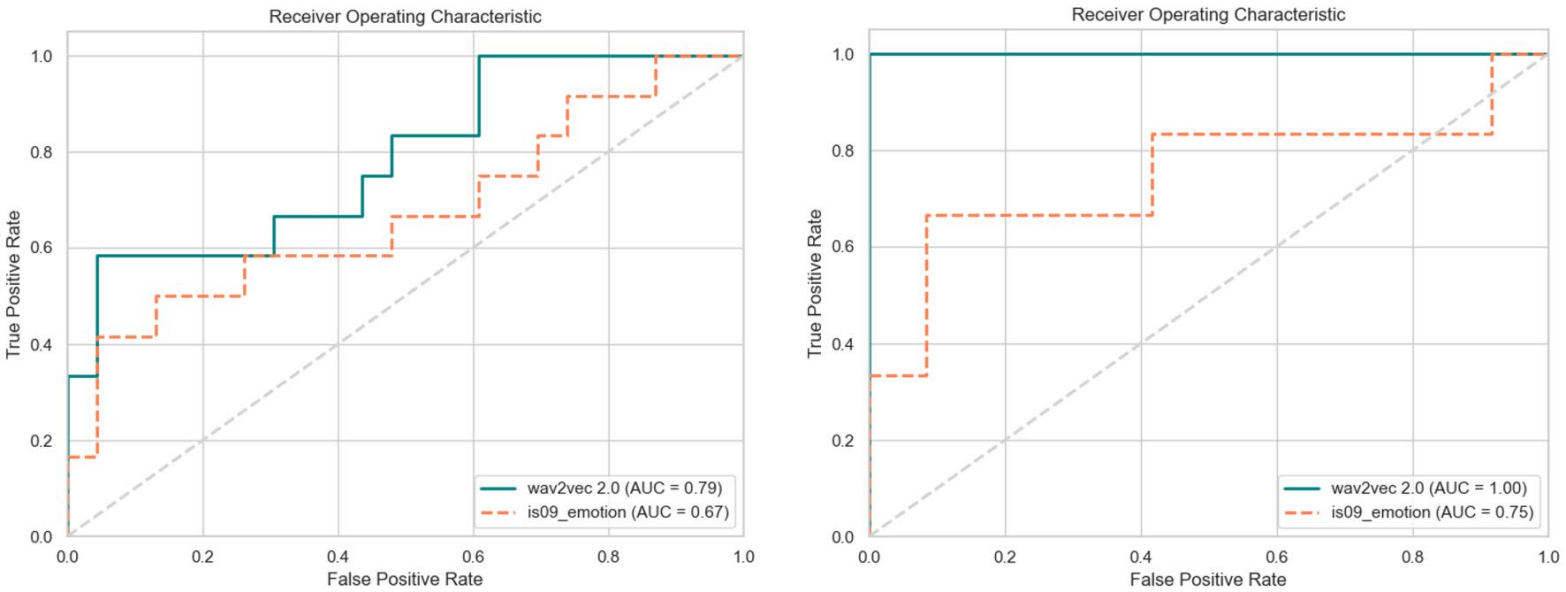
ROC curves were generated for various feature inputs using the same model. The left side represents the DAIC-WOZ dataset, while the right side corresponds to the CMDC dataset.

In this section, we further compared the binary classification performance of two different features in the same model. Through the Receiver Operating Characteristic (ROC) curve and the Area Under the Curve (AUC) metric, we evaluated the overall performance of the model. The ROC curve shows the performance of the classifier at different thresholds, where the closer the ROC curve is to the upper left corner, the better the classifier's performance. From Fig. 5, it can be seen that the fine-tuned wav2vec features are positioned more to the left and have a higher AUC value. It is worth noting that, on the CMDC dataset, although there are some misclassifications, the model performs well in terms of the AUC metric, reaching the highest value of 1, indicating that the model can perfectly rank positive instances ahead of negative instances, showing a high classification ability.

### Comparison of different acoustic features

**Figure 6:**
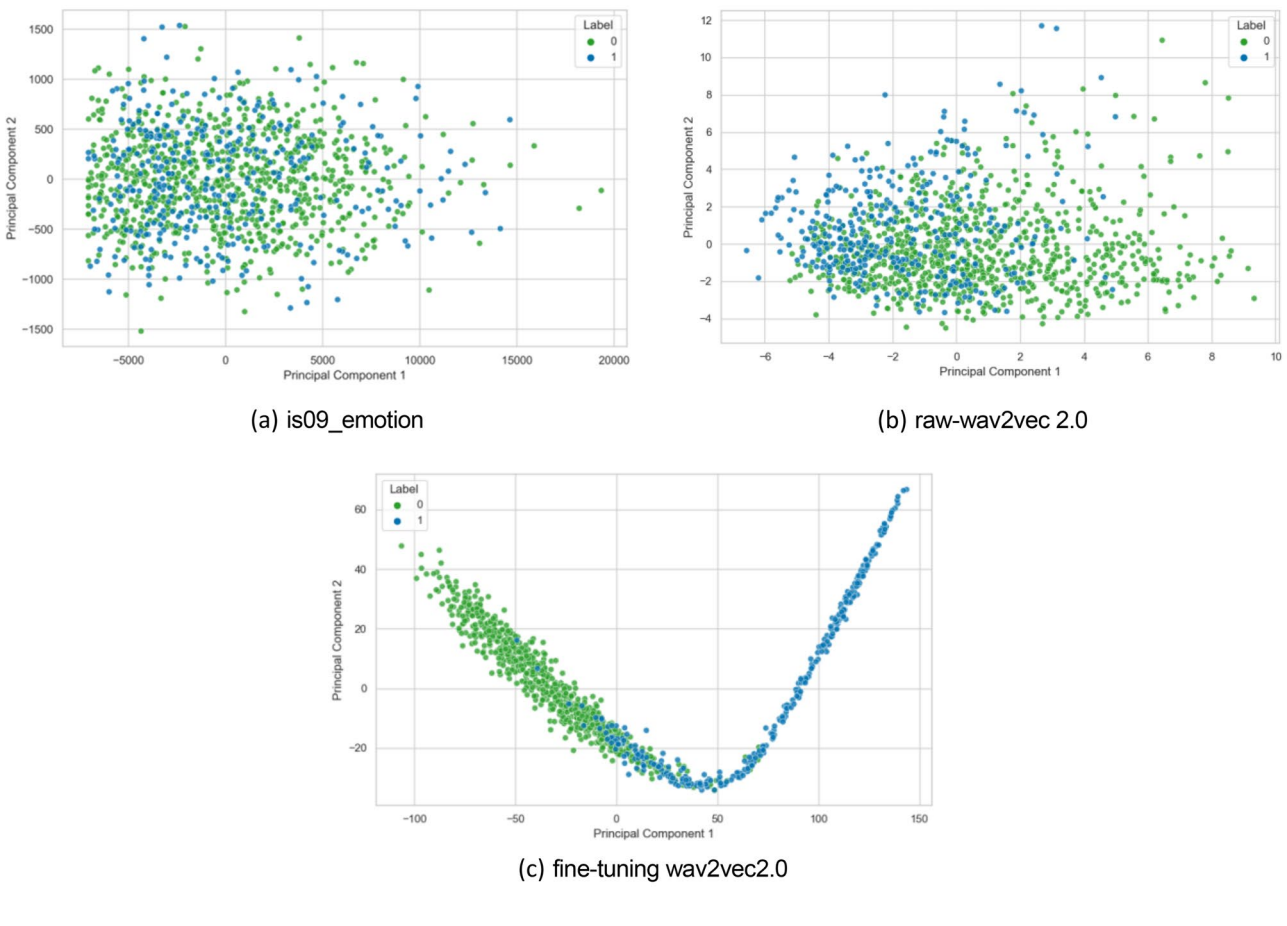
Clustering results of is09_emotion (**a**), raw-wav2vec2.0 (**b**) and fine-tuning wav2vec2.0 features (**c**).

To assess the performance of our model in acoustic feature recognition, we conducted a clustering analysis, focusing on three aspects: the is09_emotion feature set, features extracted by wav2vec2.0, and those extracted by the fine-tuned wav2vec2.0. The is09_emotion feature set offers abundant prosodic features, and the clustering analysis results are shown in Fig. 6(a). As can be seen from the figure, the clustering effect is not satisfactory, with blurred boundaries between clusters, indicating that the model is unable to effectively divide the data into meaningful groups. After clustering the features extracted by the raw-wav2vec2.0 model, the results are presented in Fig. 6(b). Compared to the is09_emotion feature set, there is some improvement, but still many features are incorrectly assigned to the wrong clusters. The fine-tuned wav2vec2.0 achieved significant improvement in feature clustering, and the results are shown in Fig. 6(c). We observed that the feature points clustered into two tightly connected groups, with distinct boundaries between them. This indicates that the fine-tuned wav2vec2.0 model demonstrates enhanced speech representation capability, effectively distinguishing features between individuals with depression and healthy controls.

### Ablation analysis

In this section, we perform a thorough validation of each module's functionality through an ablation study of the model modules. The ablation experiments are conducted with a consistent setup, where configurations remain uniform, and variations are constrained to the modules under scrutiny.

#### Comparison of fine-tuning strategies on depression detection performance

In this section, we meticulously compare the performance of fine-tuned and non-fine-tuned models in the task of speech-based depression detection through A and B experiments. Experiment A employs a pre-trained model without fine-tuning on depression speech data, whereas Experiment B incorporates fine-tuning on the depression speech dataset. This design aims to assess the effectiveness of domain-specific fine-tuning and the direct application of pre-trained models in the target domain. The experimental results are presented in Table 3.

**Table 3:** Comparison of fine-tuning strategies on depression detection performance.

	Num_layers	Pretrained_model	Precision (%)	Recall (%)	F1-score (%)
A.frozen	The last layer	wav2vec2-base	68.00	66.30	66.86
		wav2vec2-large	68.30	68.30	68.30
		wav2vec2-IEMOCAP	83.82	54.17	48.04
B.fine-tune	The last layer	wav2vec2-base	64.32	62.14	70.86
		wav2vec2-large	75.00	72.64	73.48
	All layer	wav2vec2-base	88.33	70.83	72.81
		wav2vec2-large	84.49	76.99	79.00

Foremost, it is crucial to highlight that the A and B experiments demonstrate a noteworthy performance improvement in fine-tuned models compared to non-fine-tuned models. This aligns with expectations, indicating that fine-tuning more effectively captures depression-related speech features, thereby enhancing performance in the task of SDD. Additionally, our observation reveals that the large model outperforms the base model, likely owing to its increased parameter count, allowing for a more comprehensive learning of features in the target domain and subsequently improving depression detection accuracy. This observation is consistent with the prevailing perspective in the field of deep learning, where larger models typically exhibit better performance on complex tasks. Furthermore, we note that the wav2vec 2.0 model, when fine-tuned on the IEMOCAP emotional analysis dataset, using the last layer as feature input, demonstrates good precision but relatively lower recall and F1 score. This underscores the significance of fine-tuning in the depression detection task to more effectively adjust to the speech expression features of the target domain and enhance model performance. Finally, the results suggest that, within the fine-tuning strategy, fine-tuning all layers surpasses the performance of fine-tuning only the last layer. This indicates that, in the depression detection task, adjusting features at deeper levels more comprehensively captures depression-related information in speech data. In contrast, fine-tuning only the last layer may not sufficiently capture domain-specific features, thus limiting performance improvement.

#### Comparison with different pooling strategies

**Figure 7:**
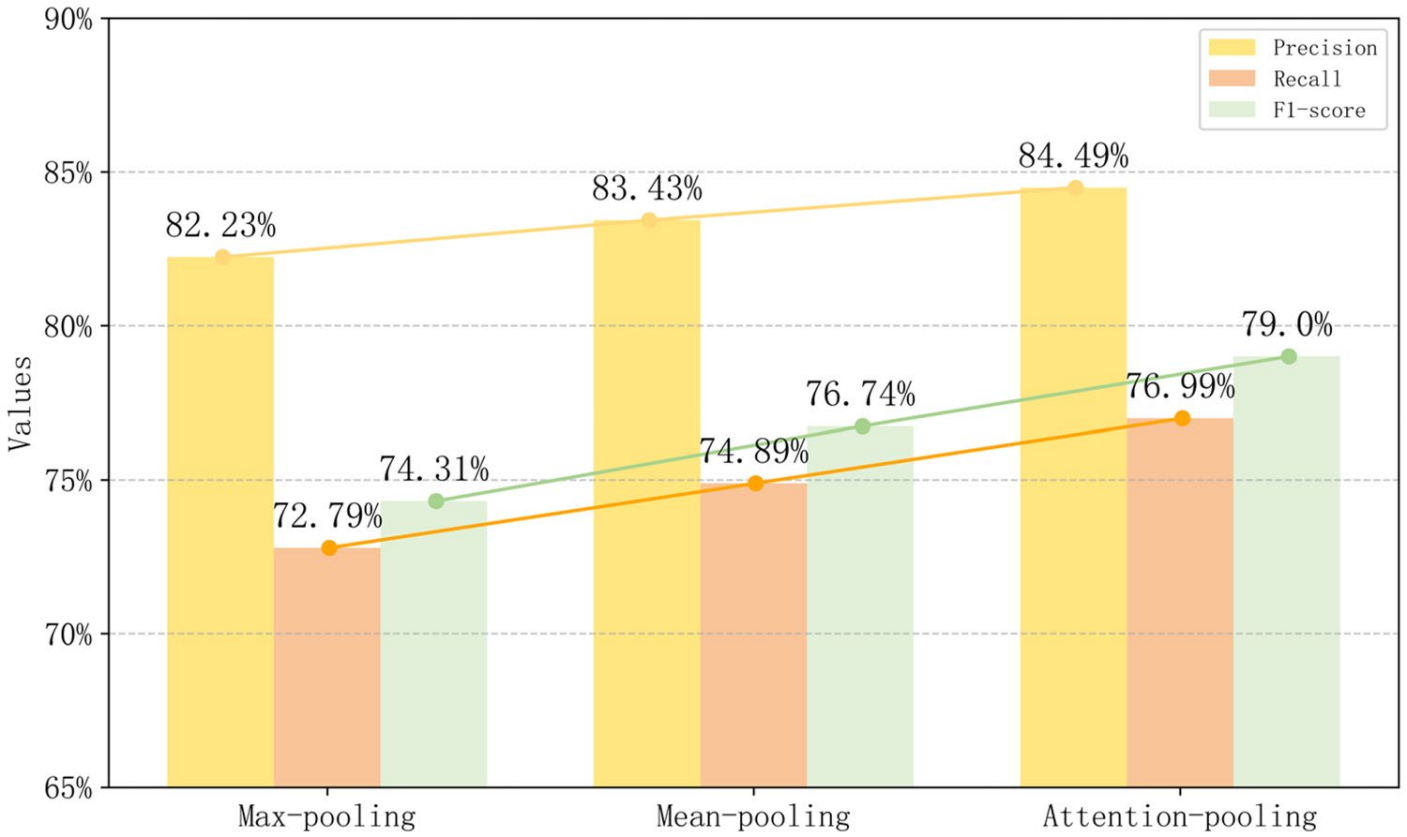
The impact of different pooling methods on model performance.

In addition to fine-tuning, we extended our investigation to compare various pooling strategies. Figure 7 illustrates that attention pooling outperformed max pooling and average pooling in F1 score by 4.69% and 2.26%, respectively. While average pooling has been proven effective in capturing features of the entire speech segment, and max pooling is adept at highlighting the most prominent features within the segment, attention pooling demonstrated superior performance. Unlike average pooling, attention pooling facilitates the model in focusing on important frame information within speech segments, contributing to enhanced model accuracy. In the context of depression detection, a more comprehensive consideration of speech segment information is shown to contribute to improved model performance.

#### Comparison with and without self-attention mechanism

To evaluate the effectiveness of the self-attention module in selecting valid information in speech segments, we conducted an ablation study by excluding this module from our proposed method. Specifically, in the absence of the self-attention module, we utilized the output of the last step of the LSTM model and connected it to a fully connected classification layer to obtain the classification result. Table 4 illustrates that the model integrated with self-attention surpasses the performance of the model lacking self-attention. This outcome suggests that, in the task of speech-based depression detection, emotional expression may concentrate in specific speech segments, and the self-attention mechanism proves more effective in capturing these crucial pieces of information.

**Table 4:** Comparison between the model with and without self-attention mechanism.

Method	Precision	Recall	F1-score
Without self-attention	82.14%	72.83%	74.72%
With self-attention	84.49%	76.99%	79.00%

#### Comparison of different audio lengths

**Figure 8:**
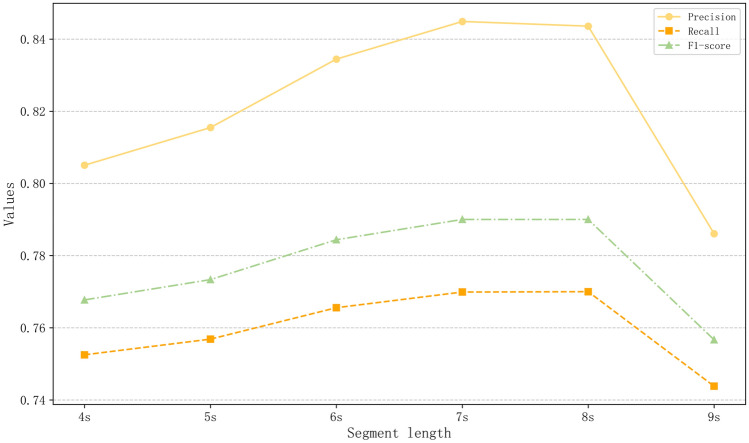
Comparison of performance across different segment lengths.

We selected the analysis of audio segments between 4 and 9 s to explore the influence of different audio lengths on model performance. This range is a commonly used segmentation method in current literature. For each segmentation strategy, we applied the aforementioned fine-tuning method and the optimal model structure for validation. Each segmentation experiment was repeated 5 times, and the averages were taken. The experimental results are depicted in Fig. 8. It is observed that with the increase in audio segment duration, the model performance shows an upward trend before 7 s, reaching a performance plateau around 7 and 8 s. This suggests that shorter speech segments may disrupt the continuity of emotions, while excessively long segments may result in insufficient sample quantity. Considering the impact of audio length on the overall sample size and model computational efficiency, we selected 7 s as the optimal duration.

#### Discussion and limitation of our work

In this study, we conducted an extensive exploration of the potential application of the audio pre-training model wav2vec 2.0 in the context of SDD. Through comparisons with traditional methods, we validated that the wav2vec model, after transfer learning on tasks with limited speech data, significantly outperforms traditional acoustic feature representations, demonstrating advanced feature representation. This underscores the feasibility of employing speech-based depression detection in low-resource scenarios. Moreover, our implementation of ablation experiments unveiled a critical insight: not all depressed patients exhibit obvious depressive characteristics in their speech, emphasizing the necessity of extracting key information from dialogues. Concurrently, we observed that traditional feature representations often overlook the temporal relationships between frames. To address this, we introduced an attention pooling structure, which, in comparison to traditional statistical functions, more effectively captures the temporal relationships between frames, yielding more expressive sentence-level vector representations for downstream tasks.

Despite these advancements, our work is not without limitations. Firstly, the integration of multiple acoustic features remains an area for improvement. While our study generates depression acoustic features based on wav2vec through transfer learning, the potential benefits of effectively fusing various acoustic features to enhance model performance and robustness cannot be overlooked. Secondly, the real-time aspect of depression detection systems requires addressing. With the prevalence of smart devices and the Internet of Things, future research should prioritize the advancement of real-time speech analysis systems for immediate and personalized depression risk assessment. The key challenge lies in achieving the real-time deployment of complex machine learning technologies^45^, such as large pre-trained models like wav2vec 2.0. We must explore embedding these large models into real-time analysis solutions and ensure their effectiveness in real-time environments through adaptive data transformations. Solving this issue is crucial for the practical application of depression detection technology in real-world scenarios.

### Conclusion and future work

In the realm of speech-based depression detection, this study has yielded significant results through thorough research and optimization of the wav2vec 2.0 model. The comparison between fine-tuned and non-fine-tuned models revealed that fine-tuned models excel in capturing speech features related to depression, consequently enhancing detection performance. Particularly noteworthy is the finding that, within the fine-tuning strategy, fine-tuning all layers surpasses the performance of fine-tuning only the last layer, underscoring the importance of adjusting features at a deeper level to adapt to the task. Regarding model structure, our exploration of different pooling strategies indicated that attention pooling achieves a higher F1 score compared to max pooling and average pooling. The incorporation of attention mechanisms proved instrumental in enhancing model accuracy. Furthermore, the ablation study confirmed the efficiency of the self-attention module in capturing key information within speech segments. This study not only provides guidance for the task of SDD but also imparts valuable experience and insights for employing deep learning in the domain of speech emotion analysis. Our work has not only achieved superior performance in acoustic feature extraction but has also presented an effective approach to address the issue of data sparsity.

Future endeavors will delve into exploring more effective feature extraction methods and strive to integrate multiple acoustic features efficiently, thereby further improving the accuracy and robustness of speech-based depression detection. Additionally, efforts will be directed towards overcoming the challenge of real-time implementation by investigating approaches such as lightweight models or employing model pruning techniques. Finally, because of the high temporal resolution, non-invasiveness, and harmlessness of electroencephalography (EEG)^46^, we plan to incorporate EEG signals into our considerations and conduct comprehensive analysis in combination with acoustic features. This approach is expected to lead to a more comprehensive and accurate depression detection method, which will provide strong support for early diagnosis, treatment, and intervention of depression, and thereby improve patients' medical experience and quality of life.

## Data Availability

The DAIC-WOZ dataset is publicly available at (https://dcapswoz.ict.usc.edu/). The CMDC dataset is publicly available at (https://ieee-dataport.org/open-access/chinese-multimodal-depression-corpus).
